# Correlation of Demographics, Healthcare Availability, and COVID-19 Outcome: Indonesian Ecological Study

**DOI:** 10.3389/fpubh.2021.605290

**Published:** 2021-02-01

**Authors:** Gede Benny Setia Wirawan, Pande Putu Januraga

**Affiliations:** ^1^Center for Public Health Innovation, Faculty of Medicine, Udayana University, Bali, Indonesia; ^2^Discipline of Public Health, College of Medicine and Public Health, Flinders University, Adelaide, SA, Australia

**Keywords:** COVID-19, demographic, healthcare availability, Indonesia, ecological study

## Abstract

**Objective:** To analyze the correlation between demographic and healthcare availability indicators with COVID-19 outcome among Indonesian provinces.

**Methods:** We employed an ecological study design to study the correlation between demographics, healthcare availability, and COVID-19 indicators. Demographic and healthcare indicators were obtained from the Indonesian Health Profile of 2019 by the Ministry of Health while COVID-19 indicators were obtained from the Indonesian COVID-19 website in August 31st 2020. Non-parametric correlation and multivariate regression analyses were conducted with IBM SPSS 23.0.

**Results:** We found the number of confirmed cases and case growth to be significantly correlated with demographic indicators, especially with distribution of age groups. Confirmed cases and case growth was significantly correlated (*p* < 0.05) with population density (correlation coefficient of 0.461 and 0.491) and proportion of young people (−0.377; −0.394). Incidence and incidence growth were correlated with ratios of GPs (0.426; 0.534), hospitals (0.376; 0.431), primary care clinics (0.423; 0.424), and hospital beds (0.472; 0.599) per capita. For mortality, case fatality rate (CFR) was correlated with population density (0.390) whereas mortality rate was correlated with ratio of hospital beds (0.387). Multivariate analyses found confirmed case independently associated with population density (β of 0.638) and demographic structure (−0.289). Case growth was independently associated with density (0.763). Incidence growth was independently associated with hospital bed ratio (0.486).

**Conclusion:** Pre-existing inequality of healthcare availability correlates with current reported incidence and mortality rate of COVID-19. Lack of healthcare availability in some provinces may have resulted in artificially low numbers of cases being diagnosed, lower demands for COVID-19 tests, and eventually lower case-findings.

## Introduction

The end of August 2020 marked the 6th month of the COVID-19 pandemic in Indonesia. Since the first confirmed case was announced in March 2020, there has been 177,571 confirmed cases, with 69,195 occurring in August alone. At the same time, there has been 7,505 mortalities with the nationwide case fatality rate from COVID-19 at 4.2% (1).

Looking closer into the distribution of these cases and mortalities, we can start to see disparities between Indonesian provinces. Disparities in health outcomes between provinces is not new in Indonesia (2), an island nation comprised of more than 17,000 islands divided into 34 provinces.

Studies have been conducted into this inequality and formulated into a public health inequality index (PHDI), which reported wide variation among Indonesian provinces, including in the healthcare provision sub-index (3). Inequality was also observed on social determinants of health, such as socioeconomic status and basic demography (2, 4).

Similar ecological studies showed that areas with lower socioeconomic status were related with higher rates of COVID-19 transmission, as well as mortality (5, 6). These results were also replicated in population-based studies (7, 8). However, few of these studies discussed how the inequality of healthcare resources and access potentially contribute to these correlations, despite previous evidence on the association between low socioeconomic income and lower healthcare access (9, 10).

In 2020, faced with a novel and global pandemic, inequality in demography and healthcare availability among provinces may contribute to different public health approaches in mitigating the pandemic and its eventual outcome (11). Other studies have reported how geographic inequalities, including socioeconomic and demographic ones, affect the COVID-19 spread rate (12, 13).

Six months into the pandemic seems to be a good time to begin evaluation on how pre-existing inequality affects the preliminary outcome of the COVID-19 pandemic mitigation in Indonesia. Thus, this study aims to explore the association between pre-existing demographics and healthcare availability variation with the current COVID-19 pandemic among Indonesian provinces.

## Materials and Methods

### Study Design

We conducted an ecological study using pre-published data on Indonesian demographic and healthcare availability. Data on demographic and healthcare availability indicators were obtained from the Indonesian Health Profile of 2019 from the Indonesian Ministry of Health website, which can be accessed from https://pusdatin.kemkes.go.id/folder/view/01/structure-publikasi-data-pusat-data-dan-informasi.html. It was an annual report on the state of the Indonesian health system, including its determinants, capacities, and outcomes (14). Meanwhile data related to the COVID-19 pandemic development in Indonesia was obtained directly from the daily updated web page of the Indonesian Task Force on COVID-19, which can be accessed from https://covid19.go.id/peta-sebaran (1). All data was collected for all 34 Indonesian provinces, with detail on 10 provinces in Sumatra and its surrounding isles, six provinces in Java, five provinces in Kalimantan (Borneo), six provinces in Sulawesi (Celebes), three provinces in Lesser Sunda Isles, and two provinces each in Maluku Isles and Papua.

### Data Collection

We collected data on demographics, healthcare availability, and COVID-19 indicators from the aforementioned data sources. Demographic indicators used in this study include population density, demographic structure, percentage of young, productive age, and elderly population), and people living under poverty. Young age was defined as those under 15 years old, productive age were 15–65 years old, and the elderly were over 65 years old. These indicators meant to reflect population connectedness, the distribution of at-risk groups, and the socioeconomic susceptibility of each province. These indicators were selected to be analyzed due to previous evidence on COVID-19 infection distribution based on age group and socioeconomic status (7, 15–17).

Health availability indicators included in this study were the ratio of general practitioners, primary care clinics, hospitals, and hospital beds. All these indicators were calculated by dividing the published number of general practitioners, primary care clinics, hospitals, and hospital beds by population size for each province, displayed as units available for 100,000 residents for general practitioners, primary care clinics, and hospitals, and units available for 1,000 residents for hospital beds. For context, primary care clinics in Indonesia include clinics, private general practitioner practices, public health centers, and basic hospital registered primary care providers in partnership with the Indonesian single payer health system. General practitioners and primary care ratios were meant to reflect provincial primary healthcare availability while hospital and hospital bed ratios were meant to reflect secondary and tertiary care availability.

COVID-19-related indicators were classified into case-finding indicators and mortality indicators. Case finding indicators include confirmed cases and daily case growth. COVID-19 cases in Indonesia are defined according to the Indonesian Ministry of Health regulation, itself referring to the WHO's recommendations, which require confirmation by nucleic acid amplification by RT-PCR procedure (18). Case growth was defined as the number of new confirmed cases found daily. Case-finding indicators also include incidence and daily incidence growth, defined as confirmed cases and case growth weighted with provincial population size.

COVID-19 mortality indicators include case fatality rate (CFR) and mortality rate. CFR was defined as percentage of mortality among confirmed cases, whereas mortality rate was confirmed COVID-19 mortality per population size.

All data on cross-sectional COVID-19 indicators (incidence, CFR, and mortality rate) were obtained on 31st August 2020. Meanwhile, for longitudinal indicators (daily case growth and incidence growth), data were collected since national case reached 100,000 on the 27th July 2020. This starting point was selected to give a more equal starting point between provinces as first reported cases and initial case-finding efforts may have differed. By this point, all Indonesian provinces had reported confirmed COVID-19 cases in their territory, a milestone passed on the 9th of April 2020.

### Data Analysis

We conducted correlation analyses between demographics, healthcare availability, and COVID-19 outcome indicators. Parametric Pearson and non-parametric Spearman correlation was conducted based on a normality test (Shapiro-Wilk) result. All analyses were performed using IBM SPSS 23.0. Statistical significancy cut off was determined at *p* < 0.05. We also conducted multivariate linear regression for variables with *p* < 0.25.

### Ethical Statement

This study analyzed deidentified publicly available data and as such was exempt from Udayana University Ethical Committee reviews.

## Results

We found wide variation between all indicators among Indonesian provinces. Based on Shapiro-Wilk test, all data were found with non-normal distribution, except for percentages of young and productive age populations, as well as the ratios for hospitals and hospital beds. Thus, all data were displayed in median with variance displayed as minimum and maximum values for each measure (Table 1).

**Table 1:** Description of median values for all indicators.

**Variable**	**Median (IQR)**
**Demographic**	
Population density	103.42 people/km^2^ (46.85 – 249.74)
Proportion of young resident	27.94% (26.53 – 29.81)
Proportion of productive age resident	67.67% (65.45 – 68.88)
Proportion of elderly resident	4.58% (3.75 – 5.54)
Residents living under poverty line	8.76% (6.30 – 13.75)
**Healthcare availability**	
Ratio of general practitioner	19.74 GPs/100,000 residents (16.11 – 26.38)
Ratio of primary care clinic	10.78 clinics/100,000 residents (8.77 – 14.91)
Ratio of hospital	1.08 hospitals/100,000 residents (0.82 – 1.31)
Ratio of hospital beds	1.21 beds/1,000 residents (1.03 – 1.53)
**COVID-19 case-finding**	
Confirmed case	1,950.50 cases (582.50 – 4,642.25)
Case growth	25.36 cases per day (7.24 – 61.01)
Incidence	49.31 cases/100,000 residents (22.45 – 112.76)
Incidence growth	0.66 cases/100,000 residents/day (0.28 – 1.03)
**COVID-19 mortality**	
Case fatality rate	3.00% (1.74 – 4.10)
Mortality rate	1.31 deaths/100,000 residents (0.50 – 4.00)

Especially wide variation was found with population density, which reflects the disparity of population concentration in Indonesia, which is concentrated on the western region, especially in provinces located in Java (six provinces) and Sumatra (10 provinces). Around 56.35% of the Indonesian population live on Java Island, which constitutes around 6.23% of her total land area. The heavily urbanized special capital region of Jakarta alone represents 3.94% of the Indonesian population, with only 0.04% of its total land area. Provinces in the western region of Indonesia also tend to have more favorable socioeconomic and healthcare availability indicators. DKI Jakarta was found to have the least poverty (3.47%) and highest availability of primary care, as well as tertiary care, with 59.49 general practitioners per 100,000 residents and 2.24 beds per 1,000 residents.

Wide variation was also found in COVID-19 indicators, both case-finding and morality related indicators. As an example, confirmed cases ranged from 177 cases in East Nusa Tenggara to 40,086 cases in Jakarta while CFR ranged from 0.52% in North Kalimantan to 7.29% in Bengkulu. Wide variation persisted after weighting by population size, which may be attributed to the aforementioned fact that population size and density also varied widely among Indonesian provinces.

Correlation analyses were then performed with a non-parametric Spearman correlation test, as all COVID-19 indicators were found to have non-normal distribution when using the Shapiro-Wilk test. Correlation results are displayed in Tables 2, 3.

**Table 2:** Correlation coefficient between demographics and healthcare availability with COVID-19 case-finding indicators.

**Variable**	**Confirmed** **case**	**Case** **growth**	**Incidence**	**Incidence** **growth**
**Demographic**				
Population density	0.461^**^	0.491^**^	−0.033	−0.006
Proportion of young resident	−0.377^*^	−0.394^*^	−0.114	−0.114
Proportion of productive age resident	0.288^+^	0.291^+^	0.211	0.228
Proportion of elderly resident	0.283	0.288^+^	−0.058	−0.089
Residents living under poverty line	−0.293^+^	−0.303^+^	−0.189	−0.197
**Healthcare availability**				
Ratio of general practitioner	0.049	0.173	0.426^*^	0.534^**^
Ratio of primary care clinic	−0.207	−0.238	0.423^*^	0.424^*^
Ratio of hospital	−0.196	−0.160	0.376^*^	0.431^*^
Ratio of hospital beds	0.172	0.284	0.472^**^	0.559^**^

+p < 0.1;

* p < 0.05;

** *p < 0.01*.

**Table 3:** Correlation coefficient between demographics and healthcare availability with COVID-19 mortality indicators.

**Variable**	**Case fatality rate**	**Mortality rate**
**Demographic**		
Population density	0.390^*^	0.183
Proportion of young resident	−0.244	−0.233
Proportion of productive age resident	0.097	0.239
Proportion of elderly resident	0.180	0.066
Residents living under poverty line	0.002	−0.150
**Healthcare availability**		
Ratio of general practitioner	0.003	0.310^+^
Ratio of primary care clinic	−0.180	0.212
Ratio of hospital	−0.300^+^	0.154
Ratio of hospital beds	0.022	0.387^*^

+p < 0.1;

* *p < 0.05*.

Absolute count of confirmed cases and case growth were found to be correlated with demographic indicators. Both measures were significantly correlated with population density. Both were also found with significant inverse correlations with the proportion of residents aged <15 years. Both measures were also correlated with almost all other demographic indicators, although with *p*-value higher than the statistical significancy threshold of 0.05. Population-weighted case-finding indicators, meanwhile, were correlated with healthcare availability. Both incidence and incidence growth were significantly correlated with ratios of general practitioners, hospitals, and hospital beds per capita.

Similar dynamics can be observed in COVID-19 mortality indicators. CFR was significantly correlated with population density and was found to have a statistically weaker correlation with hospitals per capita ratio. Meanwhile, population-weighted mortality rate indicator was not correlated with any demographic indicators, but rather was correlated with the ratio of hospital beds per capita and had a statistically weaker correlation with the ratio of general practitioners.

We conducted multivariate regression analyses for variables with *p* < 0.25 in bivariate correlation analysis. When more than one of the demographic structure indicators (proportion of population based on age groups) were eligible for multivariate analyses, only one of them would be analyzed due to the collinearity of these variables. Consequently, the strongest correlate (highest correlation coefficient) would be included in multivariate analyses while the others were excluded. Standardized coefficients (β) from regression analyses were depicted in Table 4.

**Table 4:** Standardized coefficient (β) for multivariate linear regression between demographics and healthcare availability with COVID-19 outcome indicators.

**Variable**	**Case finding**				**Mortality**	
	**Confirmed case**	**Case growth**	**Incidence**	**Incidence growth**	**Case fatality rate**	**Mortality rate**
**Demographic**						
Population density	0.638^**^	0.763^**^	N/A	N/A	−0.052	N/A
Proportion of young resident	−0.289^*^	−0.218^+^	N/A	N/A	−0.313^+^	N/A
Proportion of productive age resident	N/A	N/A	0.022	0.004	N/A	0.125
Residents living under poverty line	0.069	0.037	N/A	N/A	N/A	N/A
**Healthcare availability**						
Ratio of general practitioner	N/A	N/A	0.411	0.431^+^	N/A	0.116
Ratio of primary care clinic	−0.136	−0.102	0.259	0.129	N/A	−0.093
Ratio of hospital	N/A	N/A	−0.349	−0.330	−0.291^+^	N/A
Ratio of hospital beds	N/A	−0.009	0.399	0.486^*^	N/A	0.370

+p < 0.1;

* p < 0.05;

** *p < 0.01*.

The results bear similarity with bivariate correlation analyses. Population density and demographic structure were independently associated with confirmed cases whereas case growth was only independently associated with population density. When controlling for population size, no variable was independently associated with incidence, however, hospital bed ratio was independently associated with incidence growth. No variables were independently and significantly associated with mortality.

## Discussions

We observed a bivariate correlation between demographics, healthcare availability, and COVID-19 indicators. A correlation was observed between confirmed case count and case growth with demographic indicators that may suggest that demographic structure is indeed the main determinant of COVID-19 distribution in Indonesia. The direction of these correlations seems to be in line with what we know of COVID-19 distribution among age groups.

Interestingly, the correlation seemed to shift from demographics to healthcare availability when COVID-19 indicators analyzed were controlled for population size, for example from total case to incidence. This trend also persists in multivariate analysis although the strength of correlation, and statistical significance.

**Figure 1:**
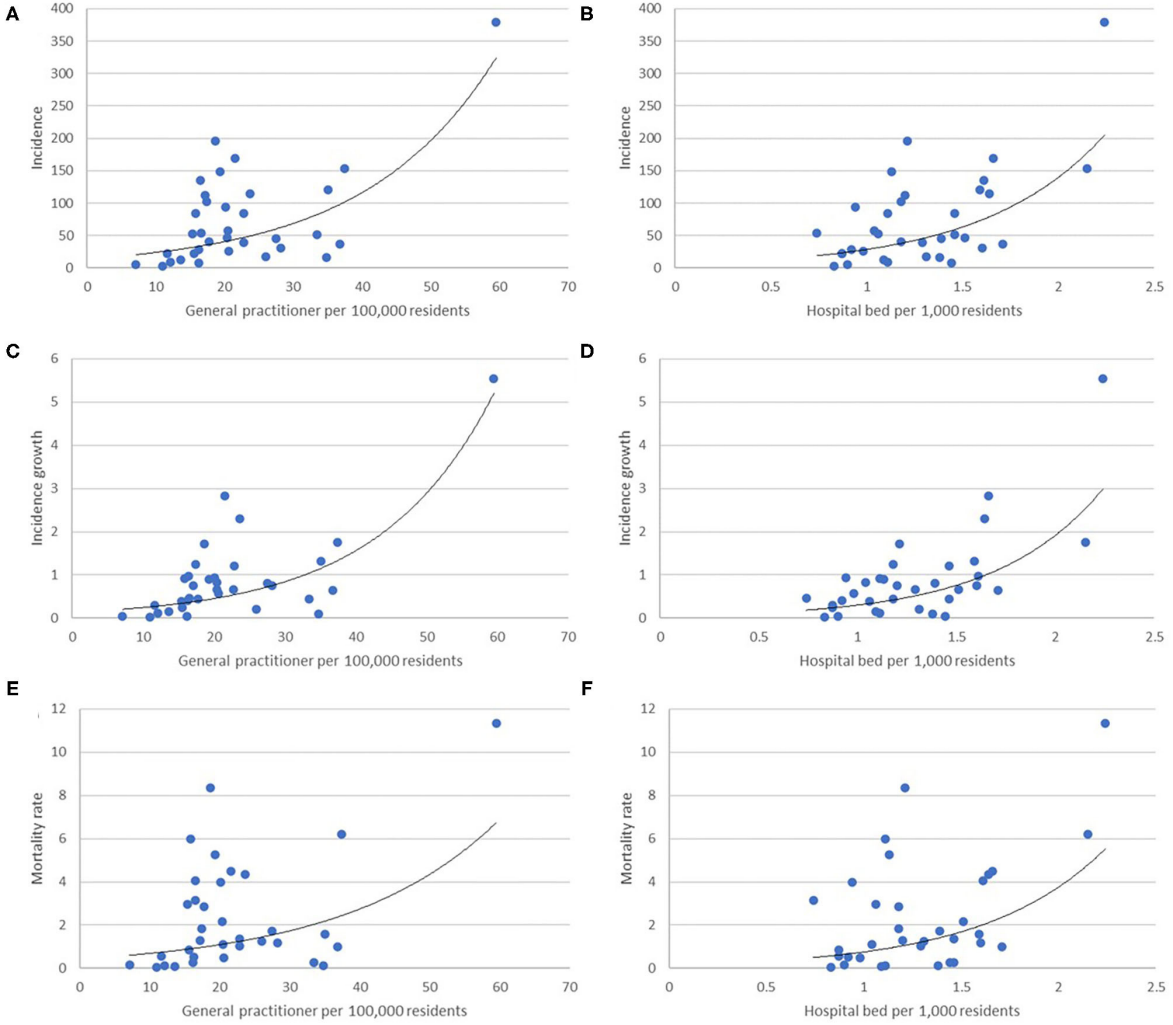
Scatter plot depicting a correlation between general practitioners and hospital beds per population, representing primary, and tertiary healthcare availability, with incidence **(A,B)**, incidence growth **(C,D)**, and mortality rate **(E,F)**.

Provinces with a high proportion of young people were found to have fewer cases and lower-case growth, while the proportion of productive and elderly populations is positively correlated with both measures, as depicted in Figure 1. This pattern of COVID-19 case distribution by age has been previously described (19) and also corroborated by Indonesian national data (1). Similar correlations have also been reported in an ecological study investigating spread rate in Northern Italy (12).

However, in our context there has been insufficient published data on the distribution of COVID-19 suspects and tests performed by age group. Public health measures implemented early in the pandemic in Indonesia suspended in-person school activities, which may limit contact with infectious people, limiting transmission. Another possibility is that children are less likely to be tested. Indeed, symptoms in children tend to be milder, which may mean that cases are misdiagnosed as other illnesses and children are not tested for COVID-19 (20).

Meanwhile, the correlation between incidence and ratio of general practitioners and beds per capita, respectively representing availability of primary and tertiary healthcare, indicates a different aspect of case detection than a simple lack of test rate. Healthcare visits, which are predisposed by healthcare availability (21), are necessary to diagnose patients as suspects of COVID-19, requiring further tests. This inequality of primary and tertiary healthcare availability comes hand in hand with testing capacity inequality. Supplementary Table 2 depicts the correlation between national referral laboratories for COVID-19 tests with primary and tertiary healthcare availability. Lack of healthcare availability would then exacerbate the problem of inadequate tests by creating an artificially low demand for COVID-19 tests, referring to the inadequate number of laboratories to perform those tests.

This result seems to corroborate suspicion that Indonesian COVID-19 data was attributed, partly, to inadequate case-finding effort. Case in point is how the current trend did not fit the earlier forecast trajectory for COVID-19 case growth despite inadequate enforcement of health protocols in some provinces (22–24).

As of the writing of this article, Indonesia has not been able to fulfill this criterion. In the last week of August 2020, daily COVID-19 test rates for suspected cases ranged from 10,000 to 20,000 tests a day, in a country with more than 260 million residents (25), much lower than the WHO recommended level of one suspected case per 1,000 population per week (26). While national referral laboratories for COVID-19 diagnosis tripled between May and July 2020 (27, 28), it still only represent <1 laboratory per 1 million residents nationwide, or a median provincial ratio of 0.84 laboratories per 1 million residents (Supplementary Table 1).

It further suggests that lack of reported COVID-19 cases in certain provinces can be attributed to an inequality of healthcare resources, both in personnel and capital investments. This condition predates the current pandemic (29) but has since became more apparent. It is further corroborated with a positive correlation between hospital beds and mortality, which indicates that a number of COVID-19 deaths may be unaccountable due to a lack of healthcare access.

We have had evidence on how inequalities affect outcomes of COVID-19. Population-based studies have reported how different age groups have different susceptibilities to transmission, either due to biologic or behavioral factors (30, 31). Poverty has also been reported to correlate with mobility and transmission (32) and lack of healthcare access has also been reported to correlate with mortality (33). Meanwhile, other studies have reported how geographical inequality leads to different COVID-19 spread and mortality outcomes (34).

However, correlation on an ecological level is frustrated by the disparity of data quality for COVID-19 indicators. A similarly peculiar pattern occurred where developed countries seemed to fare worse in COVID-19 indicators than had been previously reported (35, 36). Lack of detection availability, including low test rate, as well as alleged data manipulation by authorities has been cited as possible explanations behind these results (35).

Another interesting finding in our results is a negative correlation between poverty with confirmed case numbers and growth. This differs from previous data, both from population-based research and ecological data, which found a positive correlation between poverty and COVID-19 spread in a population (32, 37).

In an Indonesian context, there are a number of COVID-19 tests conducted by private clinics funded out-of-pocket by patients. People took these tests if they were ineligible for publicly funded COVID-19 tests or they deemed the waiting list for publicly funded tests to be too long. In this condition, residents in provinces with high poverty may be less capable of affording out-of-pocket healthcare, and thus are less likely be properly diagnosed or to afford out-of-pocket tests. Indeed, observers have reported on the unequal access to COVID-19 tests in Indonesia between different socioeconomic classes (38). However, there are insufficient data on how much these out-of-pocket tests contribute to the number of tests performed in Indonesia or in each province.

All these findings suggest that pre-existing demographics and healthcare availability in Indonesia hinders an effective response to the COVID-19 pandemic in a very basic way: it prevents policymakers from detecting the real gravity of the situation. Capacity-building efforts initiated over the course of the pandemic is much too focused on building testing capacity without much effort to build up the underlying inequality of healthcare availability. Even then, testing capacity build-up is still inadequate and needs to be accelerated.

The policy implications of these findings concern Indonesia's preparedness for future pandemics. Equalization of healthcare availability would allow policymakers to detect health problems earlier, including future outbreaks, epidemics, and even pandemics. It has been noted in another analysis of COVID-19 spread in Indonesia that increased detection capacity always led to surges in new cases, suggesting detection capacity at baseline was inadequate (39). To rectify this issue, policymakers should prioritize equalization of healthcare availability throughout the archipelago.

### Limitations of Tools and Data

We must address the validity of data from both our sources. Non-uniform eligibility for publicly funded tests between Indonesian provinces and regencies may lead to unaccounted variations in COVID-19 indicators. Regardless of an official guideline, reality in the field may be different and unaccounted for in official reports used in this study.

Data collection methods for these data sources should also be addressed. As Indonesian public agencies usually relied on bureaucratic multilevel tabulation for data gathering, there was a chance that data collected in the national level (e.g., the ones we utilized for this analysis) were not the most up-to-date version and did not accurately reflect the situation in the field. Indeed, observers have commented on discrepancies between national and district level data on COVID-19 (40) and inadequate data transparency (41). However, for the purposes of this analysis we believe it was a minor issue as the discrepancy was spread throughout the provinces.

Although multivariate analyses were performed, our study is limited in scope and scale as we only analyzed certain variable groups in association with COVID-19 outcomes with limited numbers of repetition (i.e., Indonesia's 34 provinces). Complex situations such as COVID-19 outcome may be associated with a multitude of other determinants which this study did not account for.

Our analysis may also be subject to fallacies related to drawing conclusions from ecological data. As such, population-based studies should be conducted to test the notion that the lack of healthcare access may affect the outcomes of COVID-19, from late diagnosis, late treatment, to increased risk of morbidity and mortality.

## Conclusions

We found a correlation between demographics with COVID case-findings and CFR indicators. However, controlling for population size revealed that COVID-19 incidence and mortality rate variations between Indonesian provinces to be more strongly correlated with healthcare availability in Indonesia, both at primary and tertiary levels.

Our findings corroborate the long-held suspicion that the current reported indicators of the COVID-19 situation may not reflect the real situation. Inequality in healthcare availability, which predates the current pandemic, is suggested to be a stronger determinant for the reported state of the pandemic than demographics. It put the problem in evaluating the COVID-19 situation in Indonesia to be deeper than a simple build-up of testing availability.

## Data Availability Statement

The original contributions presented in the study are included in the article/Supplementary Material, further inquiries can be directed to the corresponding author/s.

## Author Contributions

GW was responsible for conceptualization, data curation, analysis, methodology, and writing of the initial draft. PJ contributed to the writing, review, and editing process. All authors contributed to the article and approved the submitted version.

## Conflict of Interest

The authors declare that the research was conducted in the absence of any commercial or financial relationships that could be construed as a potential conflict of interest.
