# Supplementary material for: Correlation of Demographics, Healthcare Availability, and COVID-19 Outcome: Indonesian Ecological Study
Source: Front Public Health. 2021 Feb 1;9:605290. doi: 10.3389/fpubh.2021.605290 (PMC7882903; doi:10.3389/fpubh.2021.605290)
Supplement: Supplementary file 1 [file Table_1.DOCX]

Table S1. Description of availability of national referral (NR) laboratories for COVID-19 tests.

| **Variable** | **Median (IQR)** |
| --- | --- |
| NR laboratories ratio in 3^rd^ of July | 0.84 labs/1,000,000 residents (0.96) |
| NR laboratories ratio in 15^th^ of May | 0.22 labs/1,000,000 residents (0.41) |

Table S2. Correlation between laboratory availability and healthcare availability.

| **Variable** | **GP ratio** | **Primary care ratio** | **Hospital ratio** | **Hospital bed ratio** |
| --- | --- | --- | --- | --- |
| NR laboratories ratio in 3^rd^ of July | 0.352* | 0.147 | 0.285 | 0.349* |
| NR laboratories ratio in 15^th^ of May | 0.370* | -0.115 | 0.230 | 0.514* |

*p < 0.05; Note: all correlation analyses performed with Spearman non-parametric correlation test.
